# Short-Term Stability of Retinal Nerve Fiber Layer and Ganglion Cell Layer Thickness Following Direct Selective Laser Trabeculoplasty in Patients with Ocular Hypertension and Glaucoma

**DOI:** 10.3390/diagnostics16071066

**Published:** 2026-04-01

**Authors:** Dana Garzozi, Moshe Carmel, Gil Neuman, Anna Lisitsky, Zohar Bracha, Hila Givoni, Kobi Brosh, Assaf Kratz, Ahed Imtirat, David Zadok, Mordechai Goldberg

**Affiliations:** 1 Ophthalmology Department, The Eisenberg R&D Authority, Shaare Zedek Medical Center, Faculty of Medicine, Hebrew University of Jerusalem, Jerusalem 9190501, Israel; 2 Department of Ophthalmology, Barzilai University Medical Center, Ashkelon 7830604, Israel; 3 Faculty of Health Sciences, Ben-Gurion University of the Negev, Beer-Sheva 8410501, Israel; 4 Department of Ophthalmology, Soroka University Medical Center, Beer-Sheva 8410501, Israel

**Keywords:** glaucoma, direct selective laser trabeculoplasty, ocular hypertension, OCT, RNFL, retinal nerve fiber layer, GCL, ganglion cell layer

## Abstract

**Background/Objectives**: To evaluate the short-term effects of direct selective laser trabeculoplasty (DSLT) on retinal nerve fiber layer (RNFL) and ganglion cell layer (GCL) thickness in patients with ocular hypertension (OHT) and primary open-angle glaucoma (POAG). **Methods**: This retrospective, single-center study included 45 eyes of 45 patients with OHT or POAG who underwent DSLT at Shaare Zedek Medical Center between February 2024 and February 2025. The primary outcome was the change in RNFL and GCL thickness, as measured by spectral-domain optical coherence tomography (SD-OCT) before and two months after treatment. Secondary outcomes included intraocular pressure (IOP) reduction, corrected distance visual acuity (CDVA), and safety. Only high-quality OCT scans (quality score > 25) were included in the analysis. **Results**: OCT analysis revealed no statistically significant changes in the inner retinal structure two months post-treatment. The mean RNFL thickness was 77.1 ± 17.2 µm at baseline and 77.4 ± 17.3 µm at follow-up (*p* = 0.285). The mean GCL thickness remained unchanged (42.4 ± 11.6 µm vs. 42.4 ± 11.3 µm, *p* = 0.750). CDVA remained stable (0.2 ± 0.4 vs. 0.2 ± 0.4 logMAR; *p* = 0.351), and no vision-threatening complications were observed. Mean IOP decreased significantly from 19.7 ± 4.0 mmHg at baseline to 16.2 ± 3.5 mmHg at two months (*p* < 0.001). The mean total laser energy delivered was 196.5 ± 10.2 mJ (range: 176–210 mJ). **Conclusions**: DSLT was not associated with significant short-term changes in RNFL or GCL thickness, supporting its structural safety in patients with OHT or glaucoma. Further long-term studies are warranted to determine the durability of these findings and the potential neuroprotective effects of DSLT.

## 1. Introduction

Glaucoma is a principal cause of irreversible visual impairment and blindness globally, exerting a significant impact on patients’ quality of life and posing a major public health challenge in both developed and developing countries [1,2,3]. Characterized by progressive optic neuropathy, glaucoma leads to the loss of retinal ganglion cells and their axons, often silently and insidiously, until substantial visual field damage occurs. Consequently, early detection and continuous monitoring are crucial for preserving visual function, slowing disease progression, and minimizing the risk of disability. Among the available diagnostic tools, structural imaging of the retinal nerve fiber layer thickness (RNFL) and ganglion cell layer thickness (GCL) has emerged as a cornerstone for evaluating glaucomatous damage and monitoring disease progression [4,5].

In recent years, laser-based interventions have become central to the therapeutic management of glaucoma. Among these, selective laser trabeculoplasty (SLT) has gained widespread adoption as a first-line treatment for lowering intraocular pressure (IOP), particularly in patients with early stage open-angle glaucoma or ocular hypertension (OHT) [5,6,7]. SLT targets pigmented cells in the trabecular meshwork (TM), inducing a biomodulatory effect that enhances aqueous outflow without causing coagulative damage [8]. Its non-destructive mechanism has been shown to preserve the integrity of the trabecular meshwork, allowing the procedure to be safely repeated over time if the IOP-lowering effect diminishes, with a favorable safety profile [8]. Indeed, large randomized controlled trials and real-world studies have supported the efficacy, safety, and cost-effectiveness of SLT [5,6], prompting many international glaucoma societies to recommend SLT as an initial therapeutic option [6,7,9].

Despite these advantages, conventional SLT is typically performed in designated glaucoma clinics, requiring the use of a contact gonioscopy lens for precise angle visualization. This method requires a skilled operator, good patient cooperation, and precise alignment, which may not always be feasible in routine or high-volume settings [8,9]. Moreover, physical contact with the cornea may result in patient discomfort, temporary corneal haze, or microtrauma. In response to these challenges, Direct Selective Laser Trabeculoplasty (DSLT) was developed as a novel, automated, non-contact laser platform. Unlike traditional SLT, DSLT delivers laser energy through the intact sclera using a paralimbal approach, eliminating the need for gonioscopic contact [10,11,12]. The system automatically identifies the limbus and applies SLT-equivalent energy to the TM within a few seconds. DSLT is designed for greater patient comfort, simplified logistics, and the potential for wider adoption, even by non-glaucoma specialists in primary eye care settings [10]. Despite the different delivery mechanism, early clinical trials have shown that DSLT achieves comparable IOP-lowering efficacy to conventional SLT, with no increase in adverse effects [10,11,12].

Given that glaucoma is a structural disease, an accurate assessment of the neuroretinal layers is essential. Optical coherence tomography (OCT) has become an indispensable tool in this regard. As a non-invasive, non-contact modality, OCT provides high-resolution cross-sectional imaging of ocular tissues, especially suited for the retina and optic nerve head, where histological verification is impractical. This technology enables the automated segmentation and quantification of the RNFL thickness, which represents the axons of the retinal ganglion cells that course toward the optic nerve head. RNFL thinning is one of the earliest and most reliable signs of glaucomatous damage. Additionally, OCT enables the evaluation of the macular GCL thickness, which contains ganglion cell bodies and serves as a sensitive indicator of early glaucomatous involvement. Thinning of the GCL thickness, measured alone or in combination with the inner plexiform layer (IPL) as the ganglion cell complex (GCC), has been shown to correlate strongly with visual field loss and to precede changes in the RNFL thickness in some patients [13,14].

Despite the growing role of SLT in clinical practice, evidence regarding its structural effects on the inner retinal layers remains limited. Most studies investigating RNFL thickness changes following SLT have reported no significant thinning over short- and intermediate-term follow-ups, particularly in patients with early or moderate disease stages [4,15,16,17]. Nonetheless, longitudinal data suggest that slow RNFL thickness loss may still occur over time, even when IOP is adequately controlled, likely reflecting the chronic neurodegenerative nature of glaucoma [18]. These observations emphasize the need for ongoing structural monitoring, even after apparently successful laser treatment.

In contrast to the RNFL thickness changes, there is a notable lack of data on the effects of SLT or DSLT on the GCL thickness. While several studies have assessed the GCC as a whole, dedicated evaluations of isolated GCL changes remain rare. Moreover, to our knowledge, no published studies to date have evaluated short-term changes in GCL or RNFL thickness following DSLT, a treatment modality that may theoretically exert different effects due to its transscleral delivery pathway and the absence of direct angle visualization. As the clinical adoption of DSLT continues to expand, establishing a safety profile beyond IOP reduction is becoming increasingly important.

The present study aimed to fill this gap by providing the first systematic evaluation of the short-term effects of DSLT on RNFL and GCL thickness using OCT-derived metrics in a cohort of patients with primary open-angle glaucoma and ocular hypertension. Our findings offer important insights into the structural safety of this emerging laser therapy and may inform future guidelines on post-treatment monitoring strategies.

## 2. Methods

This retrospective observational study was conducted at a single tertiary care center and included patients diagnosed with OHT or primary open-angle glaucoma (POAG) who underwent DSLT during the study period. All procedures were performed between February 2024 and February 2025 at the Glaucoma Outpatient Clinic of Shaare Zedek Medical Center, located in Jerusalem, Israel. The study protocol was reviewed and approved by the Institutional Ethics Committee (Approval No. 0014-24-szmc), and the study adhered to the ethical principles outlined in the Declaration of Helsinki for research involving human participants.

### 2.1. Eligibility Criteria

To be eligible for inclusion in the study, patients were required to be over the age of 18 years and to have completed a full 360-degree DSLT treatment in a single session. In addition, all participants underwent spectral-domain optical coherence tomography (SD-OCT) imaging of the RNFL and GCL at two specific time points: once at baseline (prior to DSLT treatment) and once again at a follow-up visit two months post-treatment. To ensure data reliability and minimize image-related bias, only scans with adequate image quality, defined as an image quality score above 25 according to the manufacturer’s recommendations (Heidelberg SPECTRALIS OCT user guidelines), were included in the final analysis.

### 2.2. Exclusion Criteria

Patients were excluded from the study if they had macular pathology or optic nerve disease unrelated to glaucoma, such as age-related macular degeneration, diabetic macular edema, optic neuritis, or ischemic optic neuropathy. In addition, eyes with poor-quality OCT scans (defined as image quality score of 25 or below) or with segmentation artifacts that precluded reliable retinal layer analysis were excluded. Patients with significant media opacity, such as dense cataract or corneal haze, which could interfere with scan accuracy, were also omitted.

### 2.3. Data Collection and Imaging Protocol

Relevant clinical data were extracted retrospectively from patients’ electronic medical records. These included demographic characteristics (age, sex), ocular diagnosis, laterality, refraction, and corrected distance visual acuity (CDVA). All participants were of Caucasian ethnicity. Refractive status ranged from −0.25 to −4.50 diopters, Additionally, SD-OCT measurements for RNFL and GCL thickness were obtained for all included patients at the two pre-specified time points. Imaging was performed using the Heidelberg SPECTRALIS OCT system (Heidelberg Engineering, Heidelberg, Germany), which provides high-resolution cross-sectional images of retinal and optic nerve structures with automated segmentation features.

RNFL thickness was assessed using a 3.4 mm diameter circular peripapillary scan centered on the optic nerve head. The values were recorded both globally and by quadrants—namely superior, inferior, nasal, and temporal—allowing a topographical analysis of potential structural changes. GCL thickness was evaluated using the system’s macular analysis protocol, which covers a field of 30 degrees horizontally by 25 degrees vertically, corresponding to an area of approximately 9.0 by 7.5 mm centered on the fovea. This area includes the region most densely populated with ganglion cells and is particularly sensitive for detecting early glaucomatous damage.

### 2.4. Image Review and Quality Control

All OCT scans underwent independent quality assessment by two glaucoma specialists (D.G. and M.G.) who were blinded to the clinical outcomes of the patients. Their roles were to confirm the technical adequacy of each scan, verify the accuracy of segmentation, and ensure that only high-quality data were included in the analysis. In cases where segmentation errors were noted, the images were either corrected manually using the device software (Heidelberg SPECTRALIS OCT system software version 6.16.7.0 Heidelberg Engineering, Heidelberg, Germany) or excluded from the final dataset, depending on the severity of the artifact and its impact on measurement reliability.

### 2.5. DSLT Procedure Protocol

All DSLT procedures included in this study were performed by a single, experienced glaucoma specialist (M.G.) to ensure consistency and eliminate operator-related variability. Laser treatments were conducted using the VOYAGER^®^ system (Alcon Laboratories, Inc., Fort Worth, TX, USA), a proprietary platform specifically designed for DSLT applications. This system utilizes a frequency-doubled, Q-switched Nd:YAG laser source optimized for precise energy delivery to the trabecular meshwork via transscleral application.

The technical specifications of the laser include a pulse duration of 3 nanoseconds and a spot size of 400 μm, parameters that are tailored to achieve selective photothermolysis of the trabecular meshwork cells while minimizing collateral tissue damage. The treatment parameters were standardized across all patients for each session. A total of 120 laser pulses, each with an energy level of 1.8 mJ, were delivered at a frequency of 50 Hz. The pulses were distributed in an elliptical 360-degree pattern around the limbus, targeting the full circumference of the trabecular meshwork without requiring any contact with the corneal surface.

Prior to the procedure, all patients received topical anesthesia with oxybuprocaine hydrochloride 0.4 percent (Fischer Pharmaceuticals Ltd., Bnei Brak, Israel), instilled into the conjunctival sac to ensure patient comfort. A lid speculum was gently inserted to maintain eyelid separation and provide clear access to the ocular surface. Importantly, the procedure was performed while the patient was in a standing or upright seated position, which is a distinctive aspect of DSLT and differs from the supine sitting positioning often required in other laser or surgical interventions.

To ensure accurate delivery of laser energy, limbal localization was achieved using the system’s built-in eye-tracking module, which identifies the paralimbal zone and locks onto the treatment area. The alignment was confirmed by the operator in real time, and the laser application was conducted under direct visualization, allowing for rapid, consistent, and safe treatment delivery.

After completion of the DSLT session, IOP was measured at 30 min post-procedure using Goldmann applanation tonometry (Haag-Streit AG, Köniz, Switzerland), the clinical gold standard for IOP assessment. Patients were then prescribed topical corticosteroid therapy, consisting of dexamethasone sodium phosphate 0.1 percent (Fischer Pharmaceuticals Ltd., Bnei Brak, Israel), administered three times daily for one week to reduce post-laser inflammation. Routine postoperative instructions were provided, and patients were scheduled for a follow-up examination two months after treatment, during which both clinical assessment and structural imaging (OCT) were repeated as part of the study protocol.

### 2.6. Statistical Analysis

Descriptive statistics were employed to summarize both continuous and categorical variables. Continuous variables, including IOP, RNFL thickness, GCL thickness, spherical equivalent, and CDVA, were expressed as mean values with standard deviation (SD). Categorical variables, such as gender, eye laterality, and lens status, were presented as absolute counts and corresponding percentages.

To assess whether the distribution of categorical variables, including gender (male vs. female) and treated eye laterality (right vs. left eye), differed significantly from an expected uniform distribution, a Chi-square goodness-of-fit test was applied. This test evaluated deviations from a theoretical 50:50 distribution and helped identify any unintentional sampling biases in laterality or sex distribution.

Given that normality assumptions were not met for key outcome parameters, as determined by inspection of histograms and Shapiro–Wilk tests, non-parametric statistical methods were used. Specifically, comparisons between pre-treatment and post-treatment values from the same eyes were conducted using the paired Wilcoxon signed-rank test, which is appropriate for assessing related samples with skewed or non-normal distributions.

A two-tailed *p*-value < 0.05 was considered statistically significant for all analyses.

All statistical analyses were performed using R software (version 4.4.0), a widely validated platform for biomedical research and data science [19]. For data visualization and generation of publication-quality figures, the following R packages were used: ggplot2 (version 4.0.1), for flexible and layered plotting [20]; ggpubr (version 0.6.2), for statistical annotation and formatting [21]; and ggstatsplot (version 0.13.4), for combining plots with integrated statistical summaries and *p*-values [22].

Regarding cases in which visual acuity was recorded as “finger counting” (FC) rather than in Snellen or Logarithm of the Minimum Angle of Resolution (logMAR) notation, a logMAR equivalent of 1.90 was assigned. This conversion is based on Michael Bach’s synthesis, which aggregates values from multiple studies examining the correspondence between qualitative acuity estimates and quantitative logMAR values [23]. This method allows for consistent inclusion of low vision data in statistical summaries and group comparisons.

## 3. Results

A total of 45 eyes from 45 patients met the predefined inclusion criteria and were included in the final analysis. The study cohort consisted of 24 male (53.3%) and 21 female (46.7%) patients, with a mean age of 71.9 ± 14.2 years, ranging from 26 to 92 years, reflecting a typical age distribution for individuals undergoing treatment for ocular hypertension and early to-moderate glaucoma (Table 1).

Of the treated eyes, 28 (62%) were right eyes and 17 (38%) were left eyes, showing no significant laterality bias. In terms of lens status, 18 eyes (40%) were phakic, while the remaining 27 eyes (60%) were pseudophakic, indicating a predominance of eyes that had previously undergone cataract surgery. 

The mean total energy delivered during the DSLT procedure was 196.5 ± 10.2 mJ s, with individual values ranging from 176 to 210 mJ, consistent with the standardized laser protocol applied across the study cohort. Importantly, no intraoperative or postoperative complications were reported in any of the treated eyes. This includes the absence of transient IOP spikes, anterior chamber inflammation, corneal epithelial defects, or other vision-threatening events.

### 3.1. Structural OCT Parameters

**Figure 1:**
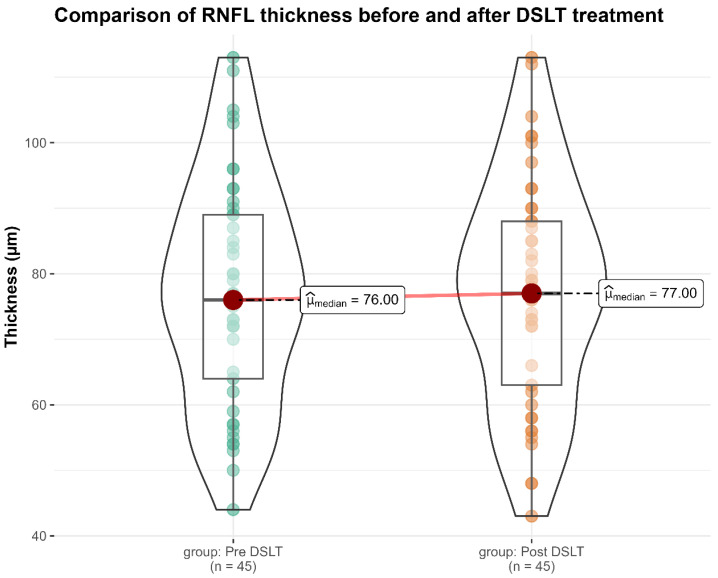
Violin plot illustrating RNFL thickness measurements before and two months after DSLT. Each violin plot represents the full distribution of RNFL thickness values at baseline and at follow-up, enabling visual comparison of structural measurements over time. The red dot indicates the median value for each time point. The thick vertical black bar corresponds to the interquartile range (IQR), capturing the central 50% of the data, while the thin black line extends to the minimum and maximum values within the dataset. The width of each violin at a given vertical point reflects the kernel density estimate, a smoothed approximation of data distribution, highlighting areas with higher or lower data concentration. This visualization allows for detection of subtle shifts in distribution beyond what is captured by summary statistics alone.

Analysis of OCT imaging revealed no statistically significant changes in RNFL thickness between baseline and the two-month follow-up following DSLT treatment. Specifically, the mean global RNFL thickness was 77.1 ± 17.2 µm at baseline, compared to 77.4 ± 17.3 µm at the two-month evaluation (*p* = 0.285), suggesting preservation of structural integrity in the short term (Table 2, Figure 1). This lack of change supports the hypothesis that DSLT, despite delivering laser energy through a transscleral approach, does not alter peripapillary axonal architecture.

**Figure 2:**
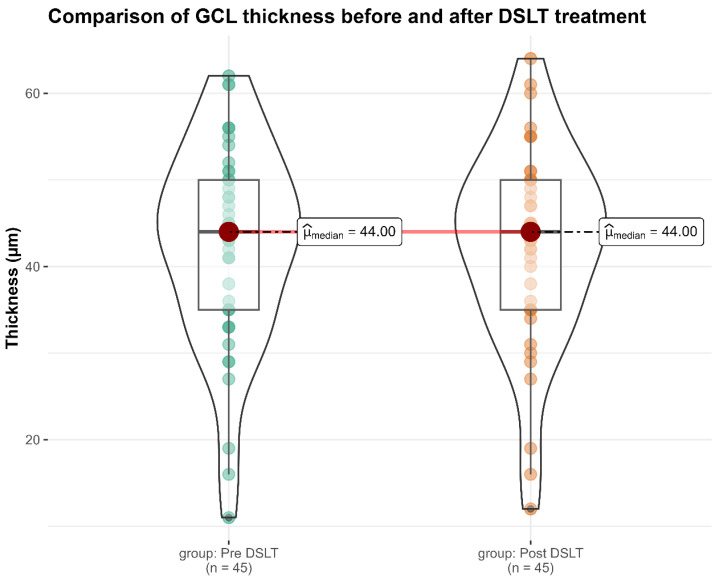
Violin plot illustrating GCL thickness measurements before and two months after DSLT. This figure presents the distribution of macular GCL thickness values at baseline and two months post-treatment. The red dot denotes the median thickness for each time point. The thick black bar represents the interquartile range (IQR), encompassing the middle 50% of observations, and the thin black line spans the entire range of measured values. The width of each violin plot reflects the kernel density estimation, offering a smoothed representation of data distribution and revealing areas with greater concentration of values. This visualization complements numerical comparisons by allowing an intuitive assessment of distributional symmetry, skewness, and the presence of outliers.

Similarly, GCL thickness remained unchanged over the same period, with a mean value of 42.4 ± 11.6 µm at baseline and 42.4 ± 11.3 µm two months after treatment (*p* = 0.750; Figure 2). This further supports the anatomical stability of the inner retinal layers following DSLT.

Subgroup analysis of RNFL thickness by quadrant (superior, inferior, nasal, and temporal) and GCL thickness by macular subfields revealed no statistically significant localized changes (Table 3). These findings suggest that DSLT does not induce focal structural damage, at least in the early postoperative period, and reinforces its safety profile with regard to neuroretinal tissue.

### 3.2. Visual Function and Refraction

CDVA and spherical equivalent (SE) were evaluated before and two months after DSLT treatment to assess potential changes in visual function and refractive status. The mean CDVA at baseline was 0.21 ± 0.39 logMAR and remained essentially unchanged at 0.22 ± 0.39 logMAR at the two-month follow-up (*p* = 0.351). Visual acuity values ranged from 0 logMAR (equivalent to 20/20 Snellen) to 1.9 logMAR, representing finger counting.

The SE showed a similarly stable pattern. At baseline, the mean SE was −1.12 ± 2.26 diopters, with a range spanning from +2.88 to −9.13 D. At the two-month visit, the mean SE was −1.21 ± 2.26 diopters, ranging from +3.00 to −8.88 D. This minimal difference was not statistically significant (*p* = 0.571), and no cases of significant post-treatment refractive shift were documented.

### 3.3. Intraocular Pressure

**Figure 3:**
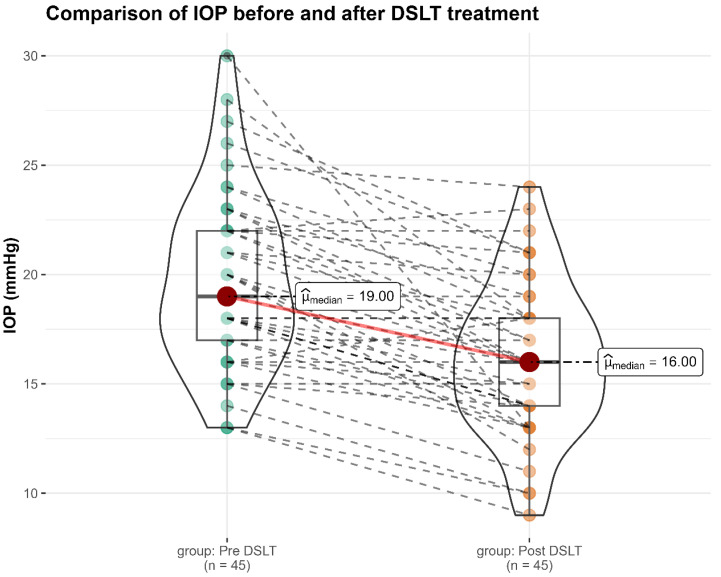
Violin plot illustrating intraocular pressure (IOP) measurements before and two months after direct selective laser trabeculoplasty (DSLT). The figure shows the distribution of IOP values at baseline and at the two-month post-treatment follow-up. Each violin represents the full distribution of IOP measurements at each time point, with width reflecting kernel density estimation. Individual eyes are displayed as colored dots, with paired measurements connected by dashed lines to illustrate within-eye changes over time. The red dot denotes the median IOP, and the box represents the interquartile range (IQR). This visualization allows assessment of changes in IOP distribution and inter-individual variability following treatment.

Intraocular pressure was measured prior to treatment and again at the two-month follow-up to assess the efficacy of DSLT in lowering IOP. At baseline, the mean IOP was 19.7 ± 4.0 mmHg (range: 14–32 mmHg). At the two-month follow-up, a significant reduction in mean IOP was observed, with a mean value of 16.2 ± 3.5 mmHg (range: 10–25 mmHg). This corresponds to a mean absolute reduction of 3.6 mmHg, reflecting an average decrease of approximately 17.3% from baseline values (*p* < 0.001, Wilcoxon signed-rank test; Figure 3). No changes in the number of topical IOP-lowering medications were observed during the two-month follow-up period (Table 4). In post hoc analysis, the IOP-lowering effect of DSLT was consistent across the study population and remained consistent when phakic and pseudophakic eyes were analyzed separately, as detailed in Table 5.

## 4. Discussion

This study is the first to evaluate the short-term impact of DSLT on the structural integrity of the RNFL and GCL using SD-OCT. These findings demonstrate that DSLT does not induce significant short-term changes in either parameter. Specifically, RNFL thickness remained stable, with values of 77.1 ± 17.2 µm at baseline and 77.4 ± 17.3 µm at two months post-treatment (*p* = 0.285). Similarly, GCL thickness showed no measurable change, with mean values of 42.4 ± 11.6 µm pre-treatment and 42.4 ± 11.3 µm post-treatment (*p* = 0.750). These results suggest that DSLT preserves the microstructural architecture of the neuroretinal tissue in the short term and supports its safety profile with regard to retinal neuronal layers.

We postulated that the mechanism of action of DSLT, like that of conventional SLT, does not adversely affect ocular structural parameters. These findings support this hypothesis, as no significant differences were observed in pre- and post-treatment OCT-derived measurements of RNFL and GCL thickness among patients with ocular hypertension and glaucoma. This observation is particularly important in the context of glaucoma, which is fundamentally an optic neuropathy characterized by progressive damage to retinal ganglion cells and their axons. Safeguarding of the integrity of the RNFL and GCL thickness is essential for maintaining visual function and represents a core objective in the management of glaucomatous disease. Any intervention that successfully lowers IOP without compromising these neuroretinal structures reinforces its role in the therapeutic armamentarium of glaucoma care.

These findings are consistent with the results of the GLAUrious randomized clinical trial [24], which demonstrated that DSLT is non-inferior to conventional SLT in lowering intraocular pressure over a six-month period, without raising notable safety concerns. Additionally, early real-world studies have further supported the efficacy and safety of DSLT, both in the short term and at one-year follow-up [11,12]. While the GLAUrious trial primarily focused on IOP-lowering efficacy and functional outcomes, this study adds a complementary perspective by specifically validating the structural safety of DSLT using OCT-derived biomarkers, namely, RNFL and GCL thickness. This contributes important new evidence to the growing body of literature supporting the clinical use of DSLT.

Furthermore, our findings are consistent with previous studies evaluating short- and medium-term RNFL changes following conventional SLT. Gedik et al. reported no significant RNFL thinning six months after SLT in patients with primary open-angle glaucoma (POAG) and OHT [25], while Lee et al. found stable RNFL thickness over a two-year period in patients with normal tension glaucoma [17]. Similarly, Wong et al. observed comparable outcomes between SLT and pattern scanning laser trabeculoplasty (PSLT) over a 12-month follow-up, reinforcing the structural safety of laser trabeculoplasty in early glaucoma management [4].

However, several longer-term studies have documented progressive RNFL thinning despite IOP-lowering interventions. Notably, Swain and Eliassi-Rad reported inferior quadrant RNFL thinning five years after SLT [26]. More broadly, longitudinal OCT studies in glaucoma have shown that progressive RNFL thickness loss may only become apparent over extended follow-up periods, beyond one or two years [27,28]. These findings underscore the importance of long-term structural monitoring to fully evaluate the neuroprotective limitations of laser-based interventions. A summary of published studies evaluating the effects of SLT on RNFL and GCL thickness is presented in Table 6.

Previous studies have suggested that macular GCL thinning may precede peripapillary RNFL loss, highlighting its potential value in the early detection and monitoring of glaucomatous damage [26,30]. However, data on isolated GCL changes following SLT remain limited. Most prior research has focused on combined segmentation of the GCL and IPL, typically reported as the GCC, without providing separate analysis of the GCL alone [31].

Notably, Kurysheva et al. [29] reported a slower rate of GCC thinning in patients who underwent SLT, suggesting a potential neuroprotective effect of the procedure. Given the established correlation between GCL thickness and visual field indices, we recommend that future studies incorporate targeted segmentation and analysis of the GCL, in order to more precisely evaluate both early glaucomatous damage and the structural safety profile of laser-based therapies.

Despite confirming the short-term structural safety of DSLT, the limited follow-up duration in this study necessitates cautious interpretation. Long-term studies of conventional SLT have shown that RNFL thinning, particularly in the inferior quadrant, usually emerge after two to five years, even under well-controlled intraocular pressure [26]. In addition, broad longitudinal OCT studies in glaucoma have demonstrated that progressive RNFL loss is most reliably detected over extended observation periods [28]. These findings likely reflect the natural progression of glaucoma and age-related neurodegeneration rather than a direct structural effect of laser trabeculoplasty.

RNFL thickness measurements may be insensitive to subtle short-term changes and are limited by test–retest variability and floor effects; therefore, complementary approaches for detecting disease progression may include electrophysiology, OCT angiography-derived microvascular metrics, and assessment of ocular perfusion pressure, which may enhance the detection of early neurodegenerative or vascular changes not captured by RNFL thickness alone [32,33,34,35].

Moreover, variability in imaging protocols, segmentation algorithms, and patient characteristics across studies may limit direct comparisons and generalizability. Therefore, standardized, long-term prospective studies incorporating quadrant-level RNFL and macular GCL thickness analysis with follow-up durations are essential for accurately assessing the long-term structural impact of DSLT in comparison to conventional SLT.

Ultimately, there remains a notable gap in the literature regarding isolated GCL analysis in the context of laser trabeculoplasty, despite growing evidence supporting its value as an early structural biomarker of therapeutic safety. Advances in OCT segmentation software may soon enable more precise and reproducible evaluations of GCL integrity following laser-based glaucoma treatments.

This study has several limitations. First, the relatively small sample size and short follow-up period may limit the generalizability of the findings and the ability to detect subtle long-term structural changes. Second, the study lacked a control group, which restricts direct comparison with conventional SLT or untreated eyes. Third, although OCT measurements were carefully reviewed, segmentation errors and variability in image quality may have influenced the results despite exclusion of low-quality scans. Fourth, the study population consisted exclusively of Caucasian patients, which may limit the applicability of the findings to more diverse populations. Finally, medication use was not formally analyzed.

In conclusion, this study provides the first OCT-based assessment of the short-term structural effects of DSLT on both RNFL and GCL thickness. No statistically significant changes were observed two months after treatment, supporting the short-term structural safety of DSLT in patients with ocular hypertension and glaucoma. Nevertheless, extended follow-up is warranted to assess the durability of these findings and to better understand the long-term impact of DSLT on retinal and optic nerve structures.

## Figures and Tables

**Table 1:** Baseline characteristics of the study population.

Variable	Value
**Number of patients**	45
**Number of eyes**	45
**Age (years)**	
** Mean ± SD**	71.9 ± 14.2
** Range**	26–92
**Sex, *n* (%)**	
** Male**	24 (53.3)
** Female**	21 (46.7)
**Eye laterality, *n* (%)**	
** Right**	28 (62.2)
** Left**	17 (37.8)
**Lens status, *n* (%)**	
** Phakic**	18 (40.0)
** Pseudophakic**	27 (60.0)
**Total laser energy (mJ)**	
** Mean ± SD**	196.6 ± 10.2
** Median (IQR)**	200.0 (192.8–204.3)

Abbreviations: SD, standard deviation; IQR, interquartile range.

**Table 2:** Structural and Functional Ocular Parameters Before and Two Months After DSLT.

Parameter	Pre-DSLT	Post-DSLT	*p*-Value
**RNFL thickness (µm)**	77.1 ± 17.2	77.4 ± 17.3	0.285
**GCL thickness (µm)**	42.4 ± 11.6	42.4 ± 11.3	0.750
**IOP (mmHg)**	19.7 ± 4.0	16.2 ± 3.5	<0.001
**IOP reduction (%)**	—	17.3 ± 13.7	—
**IOP reduction (mmHg)**	—	3.6 ± 3.3	—

Abbreviations: RNFL, Retinal Nerve Fiber Layer; GCL, Ganglion Cell Layer; DSLT, Direct Selective Laser Trabeculoplasty; IOP, Intraocular Pressure; SD, Standard Deviation; IQR, Interquartile Range.

**Table 3:** Sectoral analysis of macular GCL and peripapillary RNFL thickness before and after DSLT.

Parameter	Sector	Pre-DSLT (Mean ± SD, µm)	Post-DSLT (Mean ± SD, µm)	*p*-Value
**GCL thickness**	S	44.25 ± 11.81	44.54 ± 12.06	0.190
	NS	44.33 ± 11.74	42.64 ± 13.00	0.009
	NI	43.98 ± 12.34	42.21 ± 13.60	0.110
	I	41.71 ± 12.46	42.12 ± 12.49	0.350
	TI	39.02 ± 13.30	41.10 ± 12.16	0.041
	TS	39.10 ± 11.47	40.95 ± 10.59	0.016
**RNFL thickness**	NS	77.56 ± 25.97	84.95 ± 28.05	0.019
	N	56.93 ± 16.99	60.16 ± 15.37	0.073
	NI	86.72 ± 25.73	89.44 ± 28.79	0.130
	TI	104.81 ± 37.95	102.35 ± 36.10	0.990
	T	64.74 ± 16.39	64.28 ± 15.01	0.780
	TS	98.93 ± 32.25	95.67 ± 29.23	0.130

Abbreviations: S, superior; NS, nasal-superior; N, nasal; NI, nasal-inferior; I, inferior; TI, temporal-inferior; T, temporal; TS, temporal-superior; RNFL, retinal nerve fiber layer; GCL, ganglion cell layer; DSLT, direct selective laser trabeculoplasty.

**Table 4:** Distribution of glaucoma type and number of IOP-lowering medications before and after DSLT.

Number of IOP-Lowering Medications	POAG (n)	OHT (n)	Total (n)
**0**	5	5	10
**1**	5	2	7
**2**	5	2	7
**3**	12	1	13
**4**	8	0	8
**Total**	35	10	45

Abbreviations: OHT: ocular hypertension; POAG: primary open-angle glaucoma.

**Table 5:** Baseline characteristics, treatment parameters, and short-term outcomes in phakic vs. pseudophakic eyes undergoing DSLT.

Variable	Phakic (n = 18)	Pseudophakic (n = 27)	*p*-Value
**Age (years)**	63.56 ± 15.67	77.44 ± 9.95	0.001
**Female, *n* (%)**	6 (33.3)	15 (55.6)	0.140
**Male, *n* (%)**	12 (66.7)	12 (44.4)	
**Left eye, *n* (%)**	5 (27.8)	12 (44.4)	0.300
**Right eye, *n* (%)**	13 (72.2)	15 (55.6)	
**Baseline VA**	0.08 ± 0.08	0.29 ± 0.49	0.026
**Post-treatment VA**	0.11 ± 0.10	0.30 ± 0.48	0.100
**Baseline SE (D)**	−1.17 ± 3.17	−1.08 ± 1.38	0.400
**Post-treatment SE (D)**	−1.11 ± 3.37	−1.27 ± 1.20	0.300
**Baseline IOP (mmHg)**	21.56 ± 4.31	18.52 ± 3.23	0.013
**Post-treatment IOP (mmHg)**	17.22 ± 3.49	15.44 ± 3.36	0.064
**IOP reduction (mmHg)**	4.33 ± 4.23	3.07 ± 2.50	0.300
**IOP reduction (%)**	18.70 ± 15.70	16.38 ± 12.47	0.700
**Laser energy (mJ)**	199.78 ± 9.01	194.31 ± 10.59	0.034
**Baseline RNFL thickness (µm)**	81.94 ± 16.86	73.89 ± 16.95	0.088
**Post-treatment RNFL (µm)**	81.94 ± 17.23	74.30 ± 16.90	0.150
**ΔRNFL (µm)**	0.00 ± 1.61	0.41 ± 2.00	0.200
**Baseline GCL thickness (µm)**	46.56 ± 8.06	39.67 ± 12.83	0.051
**Post-treatment GCL (µm)**	45.94 ± 7.45	40.04 ± 12.82	0.082
**ΔGCL (µm)**	−0.61 ± 1.33	0.37 ± 0.69	<0.001

Abbreviations: DSLT, Direct Selective Laser Trabeculoplasty; VA, visual acuity; SE, spherical equivalent; IOP, intraocular pressure; RNFL, retinal nerve fiber layer; GCL, ganglion cell layer; Δ, change.

**Table 6:** Summary of Published Studies Evaluating the Effect of SLT on RNFL and GCL Thickness.

Authors	SLT Protocol	N (Eyes)	Follow-up Duration	RNFL Change	GCL Change
Lee et al. [17]	360°, single session after washout	45 eyes with NTG	24 months	No significant progressive thinning; average remained stable	Not assessed
Kurysheva et al. [29]	360° SLT, repeatable	60 eyes with PACG after LPI 64 eyes with POAG	6 years	Slower structural progression overall	Slowed GCC thinning rate (trend analysis)
Wong et al. [4]	360°, single session	67 eyes with POAG or OHT	12 months	No significant RNFL change compared with baseline	Not assessed
Gillmann et al. [15]	360°, single session SLT	37 eyes with POAG	Up to 6 months	ONH vessel density ONH rose transiently, returned to baseline at 6 months	Macular vessel density rose transiently, returned to baseline at 6 months
Gedik et al. [25]	inferior 180°	40 eyes with OHT and POAG	6 months	No significant RNFL change	Not assessed
Swain et al. [26]	360°	42 eyes with glaucoma	3 & 5 years	Mean RNFL unchanged. significant inferior quadrant thinning	Not assessed

GCL: Ganglion Cell Layer; GCC: Ganglion Cell Complex; RNFL: Retinal Nerve Fiber Layer; ONH: Optic Nerve Head; POAG: Primary Open-Angle Glaucoma; NTG: Normal Tension Glaucoma; OHT: Ocular Hypertension; LPI: Laser Peripheral Iridotomy; SLT: Selective Laser Trabeculoplasty.

## Data Availability

The original contributions presented in this study are included in the article. Further inquiries can be directed to the corresponding author.
